# Epitranscriptomics in the development, functions, and disorders of cancer stem cells

**DOI:** 10.3389/fonc.2023.1145766

**Published:** 2023-03-17

**Authors:** Linlin Hao, Jian Zhang, Zhongshan Liu, Xia Lin, Jie Guo

**Affiliations:** ^1^ Department of Tumor Radiotherapy, The Second Hospital of Jilin University, Changchun, China; ^2^ School of Life Sciences, Department of Biology, Southern University of Science and Technology, Shenzhen, China

**Keywords:** epitranscriptomics, RNA modification, cancer stem cell, stemness, RNA-modifying enzyme

## Abstract

Biomolecular modifications play an important role in the development of life, and previous studies have investigated the role of DNA and proteins. In the last decade, with the development of sequencing technology, the veil of epitranscriptomics has been gradually lifted. Transcriptomics focuses on RNA modifications that affect gene expression at the transcriptional level. With further research, scientists have found that changes in RNA modification proteins are closely linked to cancer tumorigenesis, progression, metastasis, and drug resistance. Cancer stem cells (CSCs) are considered powerful drivers of tumorigenesis and key factors for therapeutic resistance. In this article, we focus on describing RNA modifications associated with CSCs and summarize the associated research progress. The aim of this review is to identify new directions for cancer diagnosis and targeted therapy.

## 1 Introduction

Epigenetic modifications are heritable phenotypic changes that occur without altering the nucleic acid sequence (1–3), and they include DNA methylation, histone modification, chromatin remodeling, genomic imprinting, maternal effects, gene silencing, and RNA editing (4–7). The function of epigenetic mechanisms in tumors has been extensively studied in recent decades. In contrast, epitranscriptomics has received relatively limited attention in the study of tumors. Approximately 70 years ago, scientists discovered the first RNA modification in yeast tRNA, namely, pseudouridine (Ψ) (8), which was mainly distributed on noncoding RNAs (ncRNAs), such as tRNA and rRNA. At that time, the diversity of RNA modification sites was unknown, and the occurrence of RNA modifications on almost all RNA species had not been identified. In recent years, with advances in mass spectrometry and high-throughput sequencing techniques, more RNA modifications have been discovered. For example, Li et al. (9) combined the carbodiimide metho-p-toluenesulfonate (CMCT) reaction with the biotin pull-down method to map over 1000 Ψ sites in mouse and human mRNAs and ncRNAs. Two groups independently developed similar methods, MeRIP-seq (methylated RNA immunoprecipitation followed by sequencing) and m^6^A-seq (10) (11). To briefly explain the methods, RNA is fragmented into ∼100 nt long segments and is immunoprecipitated with anti-m^6^A antibody, which results in selective enrichment of methylated RNA fragments. Eluted RNAs and input control samples are deep sequenced, and the reads are mapped to the genome. Using peak-calling algorithms, regions enriched in the immunoprecipitate relative to input samples are identified as “m^6^A peaks.” These methods allowed genome-wide mapping of m^6^A modification with a resolution of ∼200 nt, detected over 12,000 m^6^A peaks in transcripts of >7,000 genes in human cells and mouse tissues. Huang et al. (12) reported transcriptome-wide loci for m^5^C using an improved bisulfite sequencing method and a novel computational approach. Pandolfini et al. (13) and Zhang et al. (14) used chemical processing combined with a reverse transcription labeling method and antibody immunoprecipitation to identify internal 7-methylguanosine (m^7^G). To date, over 170 posttranscriptional RNA modifications have been identified, and they include abundant RNA methylation modifications and other complex modifications (15, 16). However, these experimental methods also have certain disadvantages, such as time-consuming and expensive, and they are not able to keep pace with the explosive increase of RNA sequences revealed by the rapid development of sequencing technology. Instead, computational methods can be able to provide a faster and more cost-effective way for RNA modifications site identification, which contributed a lot to the development of RNA epitranscriptome study. So far, several computational methods for predicting RNA modifications site have been reported, such as the most popular databases RMBase, Modomics, m6A2target, m6A-Atlas,m5C-Atlas (17–21). In addition, some functional tools like m6ASNP, ConsRM can be used to boost further functional studies investigating genetic variants (22, 23).

Transcriptome diversity induced by RNA modifications is considered an important mechanism that drives proteome diversity (24). Recent findings suggest that various RNA modifications, including attenuation, translocation, splicing, and translation, can affect transcriptome metabolism in a tissue-specific manner (25), and these findings have contributed to the emergence of the epitranscriptomic domain. Similar to DNA methylation and histone modification, RNA modifications represent another layer of gene expression regulation. Such modifications are observed for various forms of RNA and are involved in various life processes of cellular organisms, where RNA-modifying enzymes play a decisive role (26). Moreover, abnormal RNA modifications can affect the normal life activities of organisms and lead to the development of diseases, such as cancer, neurological disorders (27), and immune disorders (28). Continued research on RNA modifications will reveal new possibilities for the rapid alteration of gene expression following specific environmental changes.

Most malignant tumors are incurable. Over the past two decades, numerous studies have shown that tumorigenesis is centered on cancer stem cells (CSCs), which represent a small subset of cancer cells with tumor initiation capacity (29). CSCs are a population of malignant tumor cells in tumor tissue capable of self-renewal, rapid proliferation, and multidirectional differentiation potential, and they have the ability to initiate and reconstitute the tumor tissue phenotype. They can self-renew through cell division to form identical daughter cells and also differentiate into various types of daughter cells (30). CSCs are involved in cancer metastasis and recurrence and promote tumor vascularization, chemotherapy and radiotherapy resistance, and immune cell surveillance evasion. According to recent therapeutic studies, specifically targeting CSCs represents a promising therapeutic strategy. RNA modifications have been extensively studied in relation to many key features of cancer, such as evading the immune system, sustaining proliferation, avoiding growth suppression, escaping apoptosis, achieving replicative immortality, acquiring metastatic potential, promoting angiogenesis, and reprogramming metabolism (25, 31–34). However, the role of RNA modifications in CSCs has not been thoroughly studied.

In this review, we present several mechanisms of RNA modifications and a brief overview of CSCs and summarize the latest progress in understanding how these RNA modifications regulate CSCs. Moreover, we explore questions that remain to be addressed in this field and offer insights and suggestions for further research.

## 2 Regulatory machinery of the epitranscriptome: Writers, erasers, and readers

Many types of RNA modifications have been identified, such as modifications with 5−methylcytosine (m^5^C), N6−methyladenosine (m^6^A), Ψ, N1−methyladenosine (m^1^A), 2´-O-methylation (N_m_), N6,2´-O-dimethyladenosine (m^6^A_m)_, and internal 7-methylguanosine (m^7^G), uridylation, and adenosine-to-inosine (A-to-I) editing (31, 35, 36). These modifications are also present in various types of RNA, including ribosomal RNA (rRNA), transfer RNA (tRNA), messenger RNA (mRNA), and other noncoding RNA (ncRNA), and they are mediated by RNA modification proteins (RMPs), which can be divided into dynamic modification and irreversible modification proteins. RMPs are divided into enzymes that deposit RNA chemical tags, enzymes that remove these tags, and enzymes that recognize these tags, which are referred to as “writers,” “erasers,” and “readers,” respectively.

### 2.1 N6-Methyladenosine (m^6^A)

**Figure 1:**
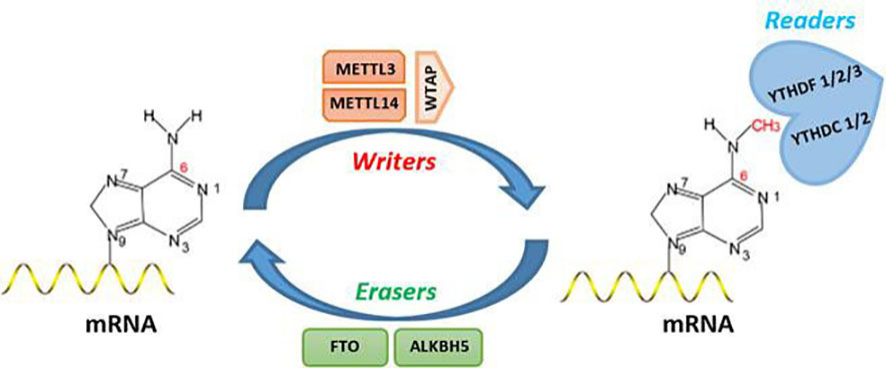
Molecular mechanism of m6A methylation modification.

As the most abundant modification in eukaryotic mRNAs, m^6^A plays an important role in regulating the processing of mRNAs (37). It accounts for more than 80% of RNA base modifications and is commonly found in different species (11, 38–40). The development of RNA immunoprecipitation sequencing (RIP-Seq) technology has led to increasing attention to m^6^A modifications in recent years (10). The m^6^A modification presents a dynamic and reversible process within cells (**Figure 1**).

#### 2.1.1 m^6^A methyltransferases—writers

The m^6^A modification is catalyzed by the methyltransferase complex, which consists of the methyltransferases METTL3 and METTL14 and its cofactors WTAP, RBM15, RBM15B, HAKAI, VIRMA (KIAA1429), and ZC3H13 (41–45). METTL3 selectively induces the methylation of the RNA motifs GAC and AAC, while METTL14 selectively induces the methylation of the RNA motif GAC. METTL3 and METTL14 are both required for the induction of m^6^A methylation (41, 46). However, the other components of the complex lack RNA methyltransferase activity. WTAP promotes m^6^A methylation by recruiting METTL3 and METTL14 to nuclear patches (47). RBM15 and RBM15B can bind to METTL3 and WTAP, and they can be localized to specific RNA sites for m^6^A modification. VIRMA preferentially mediates mRNA methylation near the 3′-untranslated region (UTR) and the stop codon (44). ZC3H13 and WTAP synergistically regulate m^6^A methylation in the nucleus (43, 45).

#### 2.1.2 m^6^A demethylases—erasers

The m^6^A modification is reversible. The removal of the methyl group from m^6^A can be achieved *via* active demethylation by the demethylase FTO and the FTO homologue ALKBH5. To date, only these two proteins (FTO and ALKBH5) have been identified as having demethylase activity (48, 49), and they both belong to the ALKB family of Fe(II)/α-ketoglutarate-dependent dioxygenases. FTO is the first enzyme identified to induce RNA demethylation (50), and it is mainly concentrated in the brain and adipose tissue (51). Compared with the tissue-based expression of FTO, ALKBH5 is highly expressed in the testis (49). These findings suggest that different demethylation enzymes may be involved in different biological processes. Consistent with the specificity of expression of these enzymes in different tissues *in vivo*, FTO knockout mice exhibit phenotypes that include increased postnatal mortality and reduced body weight (52), whereas ALKBH5 knockout mice showed impaired male fertility [43]. In addition, these proteins interact with different protein ligands to catalyze substrates of different tissues (53).

#### 2.1.3 m^6^A recognition proteins—readers

m^6^A modifications perform biological functions by binding to m^6^A “readers” containing the YTH structural domain, and these readers mainly include YTHDC1/YTHDC2, YTHDF1/YTHDF2, YTHDF3, and insulin-like growth factor (IGF)2BP1/IGF2BP2/IGF2BP3. YTHDC1 can bind to m^6^A-modified pre-mRNAs and promotes exon packaging, splicing, and mRNA export from the nucleus to the cytoplasm by recruiting the splicing factor SRSF3 and preventing SRSF10 from entering the nuclear spot (54–56). YTHDC2 selectively binds m^6^A modifications of specific motifs, thereby increasing the translation efficiency of mRNAs and decreasing the abundance of target mRNAs (57, 58). YTHDF2 and YTHDF3 can promote mRNA degradation by recognizing and binding to the m^6^A site of mRNA. In particular, the C-terminal YTH domain of YTHDF2 and YTHDF3 can bind to the m^6^A site *via* the conserved G(m^6^A)C core motif, whereas the N-terminal domain is responsible for localizing to the RNA decay site and promoting the formation of protein−mRNA complexes. Knockdown of YTHDF2 and YTHDF3 leads to a significant increase in mRNAs with m^6^A modification in cells (59, 60). At the level of mRNA translation, recognition and binding of m^6^A by YTHDF1 and YTHDF3 promote protein synthesis. YTHDF1 enhances the translation efficiency of m^6^A-modified mRNAs mainly by interacting with the translation initiation factors eIF3 and eIF4A3, whereas YTHDF3 promotes the translation of m^6^A-modified mRNAs by binding to YTHDF1 and eIF4A3 (60, 61). The m^6^A modification in 5′-UTRs increases in response to cellular stress, and m^6^A residues in 5′-UTRs promote mRNA translation by binding directly to eIF3. This process does not depend on YTHDF1 (62). IGF2BP1/2/3 differs from YTH domain-containing proteins. It recognizes the GG(m^6^A)C sequence through the K homology domain and enhances the stability of its target mRNA in an m^6^A-dependent manner under normal and stressful conditions (63).

YBX1 is a multifunctional RNA-binding protein that can act as a “reader” for m^5^C, which is crucial in regulating the survival of myeloid leukemia cells and the occurrence and development of AML. Recently, YBX1 was found to act synergistically with IGF2BPs to stabilize m^6^A-tagged mRNAs, which in turn regulate MYC and BCL2 expression levels in AML cells. This may provide a theoretical basis for therapy targeting YBX1 in myeloid leukemia (64) and could demonstrate its role in m^6^A modification.

### 2.2 N6,2´-O-dimethyladenosine (m^6^A_m_)

In addition to m^6^A, another reversible modification is observed in higher eukaryotes called N6,2’-O-dimethyladenosine (m^6^A_m_). The 5’ end of eukaryotic mRNA usually consists of a 7-methylguanosine (m^7^G) cap. The first nucleotide after the m7G cap can be methylated on the ribose. If this first nucleotide is 2-O-methyladenosine (Am), then it can be further methylated at its N6 position to produce m^6^A_m_. Reports have indicated that the initial adenosine is modified as m^6^A_m_ in 50–80% of mammalian mRNA. Moreover, this modification plays a crucial role in RNA splicing (65), small nuclear RNA biogenesis (66), mRNA stability (67), and cap-dependent translation (68).

Several studies have shown that cap-terminated m^6^A_m_ is catalyzed by phosphorylated CTD interacting factor 1 (also known as cap-specific adenosine methyltransferase, CAPAM) (68–71). Chen et al. (65)demonstrated that METTL4 can mediate the formation of m^6^A_m_ within U2 small nuclear RNA. The demethylase FTO was shown to be an “eraser” of the m^6^A_m_ modification. For example, when Mauer et al. (72) tested whether m^6^A_m_ was a substrate for FTO, they found that FTO did target m6Am and had very high catalytic efficiency. They later confirmed that the catalytic activity of FTO for m^6^A_m_ was nearly 100 times higher than that for m^6^A (72). Other studies have confirmed that mRNA-decamping enzyme 2 is the reader of m^6^A_m_. m^6^A_m_ can stabilize mRNA by preventing decamping enzyme 2-mediated decapping and microRNA-mediated mRNA degradation (67). Mauer et al. (67) suggested that m^6^A_m_ makes mRNA less susceptible to decapsidation and significantly contributes to mRNA stability. Thus, m^6^A_m_ is an RNA modification that is important for mRNA stability.

### 2.3 5-methylcytosine (m^5^C)

Although cytosine methylation has been described as a major epigenetic marker and often occurs in the CpG region of eukaryotic DNA (73), previous studies have reported that m^5^C is related to the nucleotide export (74) and translation efficiency (75) of certain target RNAs. However, the general regulatory function of m^5^C on gene expression and its precise mechanism need to be further studied. The deposition pattern of m^5^C on RNA is enriched at CG dinucleotides near the start site of mRNA transcription (73). In eukaryotes, the functions of two key writers, DNMT2 and NSUN2, in relation to m^5^C have been investigated. DNMT2 was initially identified as a DNA m^5^C methyltransferase in eukaryotic cells, although later studies showed that it functions mainly as an RNA m^5^C methyltransferase, which mainly affects the stability and biogenesis of tRNA (76) In contrast, NSUN2 has a wider range of target specificity, including long noncoding RNA (lncRNA), mRNA, and other small regulatory RNAs (such as dome RNA, 7SK, and Y-RNA) (73), and it does not overlap with DNMT2. Three recent studies published in *bioRxiv* almost simultaneously found that NSUN6 is a new type of mRNA m^5^C methyltransferase. NSUN6 and NSUN2 play a role in the nonoverlapping m^5^C site of mRNA, and they participate in almost all m^5^C modifications of mRNA (77, 78).

Recently, m5C recognition proteins have also been identified, including ALYREF and YBX1 (74–79). ALYREF is a kind of mRNA export factor that can recognize mRNA modified by m^5^C and promote its nuclear export (80). YBX1 (Y-box binding protein) is a reader of m^5^C in the cytoplasm and can regulate the stability of its target by recruiting ELAVL1 (mRNA stability maintenance protein). In human bladder urothelial carcinoma, YBX1 targets the m^5^C site on the 3’-UTR of the oncogene *HDGF* to stabilize its mRNA and provide a carcinogenic function (79). In zebrafish embryogenesis, YBX1 maintains the stability of m^5^C by recruiting Pabpc1a to C-modified maternal mRNA to regulate the transition from mother to zygote (81).

### 2.4 Pseudouridine (ψ)

Pseudouridine (also called ψ) is the most abundant modification in RNA and was also the earliest modification identified (82). With updated detection methods and techniques, researchers found that ψ modified almost all RNA (83). The isomerization of uridine to ribonucleic acid can improve the base accumulation of RNA by forming additional hydrogen bonds, thus affecting the secondary structure and changing the stability of RNA (84). Pseudouracil usually has a strong effect on different aspects of the cellular process. This base modification is catalyzed by pseudouracil synthase (PUS), which acts on the substrate through two different mechanisms. One of these mechanisms is to guide RNA-dependent Ψ acidification, in which H/ACA box snoRNAs interact with the target RNA through a specific sequence (85). Furthermore, pseudouracil can also catalyze the specific target RNA directly through an independent PUS (86). Each enzyme has a unique specificity for its target RNA and modifies uridine in a common sequence.

### 2.5 N1-methyladenosine (m^1^A)

The nucleotide modified by methylation at the N1 position of adenosine is m^1^A, which was initially found only in tRNA and rRNA (87). Its deposition in tRNA occurs in all processes of life. Recently, it was also found to be present in mRNA (88). However, different studies have come to different conclusions about the overall amount of m^1^A among mRNA modifications (31). In humans, m^1^A has been detected at positions 9 and 58 of tRNA in the cytoplasm and mitochondria and at position 1322 in the 28S subunit of rRNA (89, 90). The modification of m^1^A at position 58 in tRNA is dynamically reversible. Its methylation transferase complex is composed of TRMT6 (tRNA methyltransferase noncatalytic subunit 6) and TRMT61 (tRNA methyltransferase catalytic subunit 61) (91). ALKBH1 and ALKBH3 can act as demethylases to remove the m^1^A modification (92, 93). Moreover, YTHDF2 has been shown to bind to m^1^A with low affinity. Therefore, it is considered a potential m^1^A reader in cells (94). At present, studies on m^1^A are still very limited, which is mainly because of the low abundance of m^1^A (87) and technical limitations.

### 2.6 Adenosine-to-inosine editing

A-to-I is a type of RNA editing that is commonly observed in repetitive sequences in mammals (95). Its editing enzymes mainly include ADAR1 (adenosine deaminase acting on dsRNA 1), ADAR2, and ADAD3, and its editing activity is mainly exerted by ADAR1 and ADAR2. A-to-I is present on mRNA, tRNA, and miRNA (96), and the modification sites are also relatively abundant and include coding and noncoding regions (95). Adenosine is usually associated with uridine base pairs, while inosine has a similar structure as guanosine. Therefore, inosine produced by the A-to-I modification is misinterpreted as guanosine and, therefore, with cytosine base pairs. This may lead to alterations in the translated protein product. Since A-to-I modifications are widely present in postnatal to mature organisms and play a critical biological role in humans, it is important to map the location of editing sites to better understand the implications of this editing.

## 3 Brief overview of CSCs

The concept of CSCs was introduced in the 1970s (97). The first article demonstrating the existence of CSCs was reported in 1994 (98). CSCs usually account for a small fraction of cells in a malignant tumor, and they have the properties of stem cells. They can survive and renew themselves in their niche (99). The CSC hypothesis suggests that a lump of cancer has a hierarchical structure similar to that of normal tissue and that CSCs are located at the apex of this hierarchical tissue (100). Initially, CSCs were mainly observed in hematologic malignancies (98). With further research, CSCs were discovered in solid cancers. CSC populations have been detected in breast cancer (101), glioblastoma (GBM) (102), lung cancer (103), colorectal cancer (CC) (104), prostate cancer (105), and ovarian cancer (106).

CSCs are a population of cancer cells with the ability to self-renew and differentiate, and they are often hidden among cancers and not easily identified (107). Since CSCs are mainly derived from stem cells of the corresponding organs or tissues, stem cell markers vary from tissue to tissue (108). For example, CD44 is mainly found in CSCs of organs such as the breast, ovary, and pancreas. However, unlike normal stem cells, some markers in CSCs are associated with tumorigenic ability. CSCs have high plasticity, and the non-CSC cells surrounding CSCs also present plasticity (108). CSCs can differentiate into non-CSCs, and non-CSC cells can be dedifferentiated into CSCs and become supplemental cells. Therefore, cancer may be cured only by eliminating both types of cells.

The signals involved in the development of CSCs are numerous and include the same signaling pathways as in normal stem cells, such as the Wnt, Hedgehog, Notch, and Hippo signaling pathways (109). Other important signaling pathways in the life course that play important roles in CSCs are PI3K/Akt, MAPK, JAK/Stat, and TGF-β pathway, autophagy, and ferroptosis (110, 111). Research on small molecule drugs targeting these pathways is also ongoing. For example, clonidine, a drug approved by the FDA as an anthelmintic, is an inhibitor of the Wnt/β-catenin pathway that has also been found to inhibit CSCs in ovarian, breast, and prostate cancers and GBM (112–114). Because of the important role of CSCs in cancer development, targeting CSCs is a key strategy for cancer treatment. Research is ongoing on small-molecule drugs targeting CSCs, although these drugs have not yet been approved for use in the clinic.

CSCs have the same genetically driven mutations as most cancer cells. However, CSCs have developmental features that differ from those of non-stem cells, including epigenetic modifications and differences in gene expression profiles (30). Studies have shown that epigenetic modifications contribute to the functional heterogeneity of stem cells and maintain the hierarchy of normal tissue stem cells (115). With the development of epitranscriptomics over the last ten years, studies have shown that RNA modifications also play a role in maintaining the stemness of CSCs. Therefore, studying the role of epitranscriptomics in CSCs can help reveal small molecule drugs that be used to target RNA modifications and likely represent a therapeutic modality for eradicating tumors.

## 4 Epitranscriptomics in CSC development

RNA modification plays a variety of roles in mechanisms such as genesis of CSCs in several ways. In this section, we show the signaling pathways in which RNA modifications play a role in CSCs (**Table 1**).

**Table 1:** The main roles of epitranscriptomic factors in CSCs.

Cancer types	RNA modification	Molecular axis	Function	Molecular axis	References
Leukemia	m6A	METTL3	oncogene	METTL3/CEBPZ	121
Leukemia	m6A	METTL14	oncogene	SPI1-METTL14-MYB/MYC	122
Leukemia	m6A	FTO	oncogene	FTO/LILRB4	127
Leukemia	m6A	ALKBH5	oncogene	ALKBH5/TACC3	128
Leukemia	m6A	ALKBH5	oncogene	ALKBH5/KDM4C	129
Leukemia	m6A	YTHDF2	oncogene	YTHDF2/Tnfrsf2	132
Leukemia	m6A	IGF2BP1	oncogene	IGF2BP1/HOXB4/MYB/ALDH1AI	133
BC	m6A	ALKBH5	oncogene	HIF/ALKBH5/NANOG	137
BC	m6A	ALKBH5	oncogene	ALKBH5/KLF4	138
BC	m6A	IGF2BP1	oncogene	IGF2BP1/KB-1980E6. 3 (1ncRNA)/c-MYC	139
BC	m6A	IGF2BP2	oncogene	AURKU/IGF2BP2/DROSHA	141
BC	m6A	METTL14	oncogene	AURKU/METTL14/DROSHA	141
GBM	m6A	ALKBH5	oncogene	ALKBH5/FOXM1	144
GBM	m6A	ALKBH5	oncogene	ALKBH5/HRR/DDR	146
GBM	m6A	FTO	oncogene	FTO/ADAM19	147
GBM	m6A	METTL3	oncogene	METTL3/SOX2	148
GBM	m6A	YTHDF1	oncogene	YTHDF1/MSI1	150
CRC	m6A	METTL3	oncogene	METTL3/CBX8	157
CRC	m6A	YTHDF1	oncogene	YTHDF1/Wnt/B-catenin	159
CRC	m6A	YTHDF1	oncogene	YTHDF1/TCF7L2/TCF4	160
CRC	m6A	FTO	oncogene	B -catenin/FTO	161
HCC	m6A	YTHDF2	oncogene	YTHDF2/OCT4	163
HCC	m6A	FTO	oncogene	FTO/AMD1	164
NSCLC	m6A	ALKBH5	oncogene	ALKBH5/p53	165
PC	m6A	IGF2BP2	oncogene	IGF2BP2/DANCR (1ncRNA)	166
Bladder Cancer	m6A	METTL3	oncogene	METTL3/AFE4	167
os	m6A	unknown	unknown	unknown	168
00	m6A	FTO	anti-oncogene	FTO/PDEB4/PDE1C	169
OSCC	m6A	ALKBH5	oncogene	ALKBH5/FOXM1/NANOG	170
CSCC	m6A	METTL3	oncogene	unknown	171
CRC	m6Am	FTO	anti-oncogene	unknown	173
SCC	m50	NSUN2	anti-oncogene	unknown	176
GBM	us	PUS7	oncogene	unknown	177
HCC	mlA	TRMT6/TRMT61A	oncogene	TRMT6/TRMT61A/PPAR 8	179
OSCO	A to I	ADAR1	oncogene	ADAR1/SOX2/POUF51	180
GBM	A to I	ADAR1	oncogene	TYK2 / ADAR1 / GM2A	181
CRC	A to I	ADAR1	oncogene	ADAR1/AZIN1	183

GBM, glioblastoma; BC, breast cancer; CRC, colorectal cancer; HCC, hepatocellular cancer; NSCLC, non-small cell lung cancer; PC, pancreatic cancer; OS, osteosarcoma; OC, ovarian cancer; OSCC, oral squamous cell carcinoma; CSCC, cutaneous squamous cell carcinoma; SCC, squamous cell carcinoma.

### 4.1 m^6^A in CSCs

**Figure 2:**
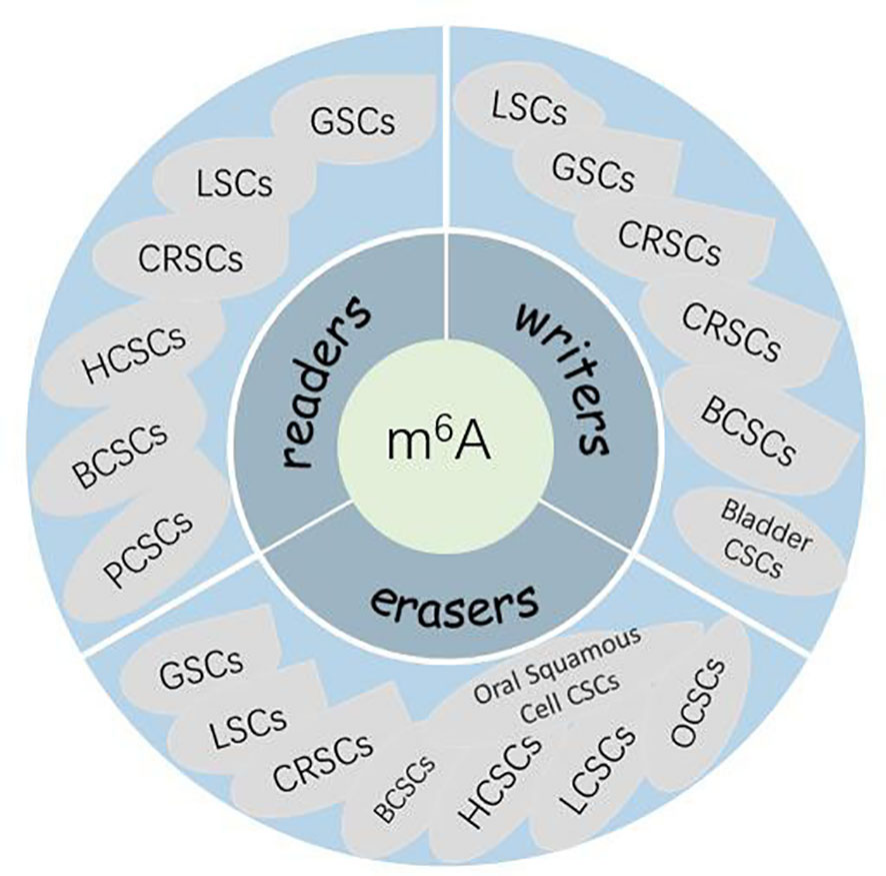
The role of m6A in maintaining CSCs.

Given the important role of m^6^A modifications of RNA in regulating gene expression and various biological processes, whether aberrant m^6^A modifications also play a role in human carcinogenesis should be investigated. It has been shown that m^6^A modifications are associated with cancer cell proliferation and differentiation, tumorigenesis, invasion, and metastasis and play an oncogenic or anticancer role in malignant tumors (116, 117). The relationship between m^6^A modifications and malignancies has been extensively reported. However, knowledge about the mechanism between m^6^A and CSC genesis is limited. CSCs are present in many myeloid leukemias and solid tumors, including GBM, breast cancer, rectal cancer, and squamous cell carcinoma of the skin. The CSC theory suggests that carcinoma initiation and growth are driven by a small number of malignant cells called CSCs. This small population undergoes continuous self-renewal to regenerate itself and differentiate into heterogeneous malignant cells, thereby initiating and maintaining tumorigenesis (118–120). A growing body of evidence suggests that CSCs may be a major cause of carcinoma resistance to conventional chemotherapy and radiotherapy (120–123). Therefore, a better understanding of the link between these stem cell-like malignant tumor cells and m^6^A modifications is necessary for developing new effective therapeutic approaches and designing new small molecules targeting CSCs. Currently, the link between RNA modifications and CSCs has also been addressed in leukemia stem cells (LSCs), breast CSCs (BCSCs), glioblastoma stem cells (GSCs), and colorectal CSCs (CRSCs). In this article, we present a comprehensive summary of studies related to m^6^A and various CSCs (**Figure 2**).

#### 4.1.1 m^6^A in hematopoietic/leukemic stem cells

Leukemia is a group of hematologic malignancies characterized by the monoclonal expansion of abnormally differentiated hematopoietic cells that can infiltrate the bone marrow and invade the blood and other extramedullary tissues. In general, leukemias can be classified as acute and chronic or gonadal and myeloid according to their progression and type of cells affected. Thus, we can identify the following subtypes: acute lymphocytic leukemia (ALL), chronic lymphocytic leukemia (CLL), acute myeloid leukemia (AML), and chronic myeloid leukemia (CML). Regarding the association between m^6^A modification and cancers of the hematopoietic system, AML is the most studied by far.

AML is an aggressive mutated disease of hematopoietic stem cells (HSCs) and hematopoietic stem progenitor cells (HSPCs), in which HSPCs present with such mutations that their myeloid differentiation is blocked, resulting in self-renewing LSCs (124). LSCs trigger leukemia. Because they are treatment-resistant, they are often considered a major cause of leukemia relapse. Therefore, there is a current clinical need to be able to identify specific therapeutic targets for the elimination of LSCs. AML is one of the malignancies with the highest expression levels of METTL3 and METTL14 (data from The Cancer Genome Atlas). Moreover, METTL3 and METTL14 were highly expressed in AML cells compared to that in normal hematopoietic progenitor cells (125–128). Consistent with the oncogenic role of *METTL3* and *METTL14* in AML, overexpression of these two genes enhances cancer cell proliferation in AML cells and progenitor cells while their downregulation strongly induces the onset of apoptosis (125, 126). In addition, the METTL3/METTL14 complex was found to promote the development of AML and maintenance of LSCs in transplanted mouse models (126, 127). In AML cells, METTL3 and METTL14 bind mainly to transcriptional start sites, although METTL3 does not always bind with METTL14. Early m^6^A cotranscriptional deposition results showed that m^6^A methylation promoted the translation of mRNAs of AML proliferation-related genes, such as *c-MYC*, *BCL2*, *PTEN*, *SP1*, and *MYB* (125–127). The transcription factor CEBPZ has an important role in hematopoietic differentiation and was shown to recruit METTL3 to the gene promoter (126).

In AML, METTL3 was shown to be localized in the cytoplasm and associated with ribosome translation. Similar results have been found in lung cancer. Furthermore, higher levels of cytoplasmic METTL3 can also lead to increased WTAP protein expression (128, 129). Since *WTAP* mRNA is not highly expressed in AML, WTAP protein levels may be associated with a concomitant elevation of the METTL3/METTL14 nuclear complex. This phenomenon is associated with carcinogenesis in AML.

Furthermore, METTL3 and METTL14 are also highly expressed in mouse and human HSCs. However, their expression is reduced when myeloid cells are differentiated. Silencing of METTL3 and METTL14 reduces stem cell proliferation and promotes bone marrow differentiation in human and mouse HSCs. Furthermore, overexpression of METTL3 or METTL14 in HSCs promoted cell proliferation and inhibited myeloid differentiation. This implies that increased m^6^A levels may alter the normal differentiation pathway in HSCs and lead to the accumulation of progenitor cells (125–128). These results suggest that METTL3 and METTL14 play a crucial role in the development of leukemia and the maintenance of LSCs.

As the first identified m^6^A demethylase, FTO has been shown to promote leukemia oncogene-mediated cell transformation as well as leukemogenesis (130). Moreover, FTO expression is significantly elevated in AML subtypes carrying MLL-AF9, PML-RARA, and FTL3-ITD translocations (131). Downregulation of FTO expression reduces the proliferative capacity of these subtypes in cell models carrying these fusion products. *In vitro* experiment results showed that FTO can promote cell proliferation/transformation, inhibit cell apoptosis, and significantly promote the occurrence of leukemia *in vivo*. Su et al. (132) found that FTO was significantly overexpressed in LSCs and showed that targeting FTO inhibited the self-renewal of LSCs. Recent studies have found that ALKBH5 is aberrantly overexpressed in AML and is essential for LSC self-renewal but dispensable for normal hematopoietic production (133). Shen et al. (133) identified its target gene, *TACC3*, in AML through sequencing and experimental verification. TACC3 is strongly associated with poor prognosis in several malignancies. Thus, the ALKBH5/m^6^A/TACC3 axis is a very promising target for therapy. Another study identified a signaling pathway involved in the critical role of ALKBH5 in AML. KDM4C (a histone demethylase) is significantly elevated in LSCs and increases the expression of ALKBH5 by increasing the chromatin accessibility of *ALKBH5* loci (134). This signaling pathway can also be used as a potential therapeutic target for LSCs.

YTHDF2 is responsible for the decay of m^6^A-modified mRNA transcripts in cells (127), which is also associated with MYC mutations in leukemia (135). Conditional knockout of mouse *YTHDF2* using the Cre/LoxP system resulted in an increased number of HSCs, thereby reducing the risk of leukemia due to abnormal HSCs (135, 136). Other findings show that the m^6^A recognition protein YTHDF2 is widely expressed in human AML and plays an essential role in the pathogenesis and metastasis of AML in mice and humans. YTHDF2 reduces the half-life of different m^6^A transcripts, including that of the tumor necrosis factor receptor TNFRSF2. These transcripts play a major role in maintaining the functional integrity of LSCs. Interestingly, YTHDF2 is not essential for normal HSC function, and its deletion does not disrupt hematopoiesis. Moreover, a deficiency of *YTHDF2* actually enhances HSC activity (137). Therefore, YTHDF2 can be identified as a unique therapeutic target. When it is reduced in expression, LSC proliferation is inhibited, and HSC amplification is promoted. IGF2BP1 is an oncoprotein expressed in various cancers. Elcheva et al. (138) found that IGF2BP1 maintains LSC properties through various stemness pathways, such as self-renewal of the key regulators of HOXB4 and MYB and regulation of ALDH1A1 (aldehyde dehydrogenase 1A1) expression.

Overall, these studies confirm the importance of m^6^A modification in LSCs, providing insights into the maintenance and self-renewal of LSCs.

#### 4.1.2 m^6^A in BCSCs

In 2021, the World Health Organization announced that breast cancer had become the most common cancer in the world, overtaking lung cancer (139). As research on breast cancer has advanced, the 5-year survival rate for patients with limited-stage tumors has reached 90%; however, the 5-year survival rate for patients with advanced-stage tumors is still less than 30%. Mortality in breast cancer is mainly due to treatment resistance and malignant metastases. New targeted therapies may help prevent initial or invasive metastases, thus improving survival and clinical outcomes in patients with advanced breast tumors. Only a small percentage of primary breast carcinoma cells have the ability to self-renew, which is an important reason for the formation of metastases or recurrence. These breast cancer cells are referred to as tumor-initiating cells or BCSCs (140).

BCSCs with stem cell properties can generate daughter BCSCs through self-renewal, thus being able to proliferate indefinitely (101, 141). They can also perform a limited number of cell divisions in a short period, producing differentiated breast cancer cells (101, 141). Reports have indicated that breast cancer cells exposed to a hypoxic environment can stimulate significantly increased expression levels of the hypoxia-inducible factors HIF-1α and HIF-2α. Then, the high expression of ALKBH5 was induced in a HIF-dependent manner, which in turn increased *NANOG* expression levels by removing methylation from the 3’-UTR of *NANOG* mRNA (142). NANOG, a pluripotent factor that maintains the properties of BCSCs, can promote the renewal of BCSCs. ALKBH5 was shown to enhance the enrichment of BCSCs in the hypoxic tumor microenvironment, and this was also demonstrated in immunodeficient mice (142). In addition to *NANOG*, ALKBH5 promotes the expression of another pluripotency gene, *KLF4*. Under hypoxic conditions, m^6^A in the mRNA of *KLF4* is demethylated to maintain the properties of BCSCs (143). Therefore, inhibiting *ALKBH5* expression by reducing NANOG and KIF4 expression is an effective strategy for targeting BCSCs *in vivo*. Moreover, hypoxia-induced lncRNA KB-1980E6.3 could recruit IGF2BP1 and m^6^A-modified *c-Myc* mRNA, which in turn increases the stability of c-Myc mRNA and maintains the stemness of BCSCs (144). m^6^A methylation may also be influenced by anticancer chemicals. Turnip sulfur, a dietary phytochemical, promotes genetic instability by reducing m^6^A methylation in BCSCs (145).

DROSHA (RNase III) is upregulated in a variety of cancers, although few studies have focused on its mechanism in promoting tumor development. Peng et al. (146) demonstrated that DROSHA cooperates with β-catenin to activate the stemness gene *STC1* and maintain BCSC properties. They found that AURKU (Aurora kinase A) kept *DROSHA* mRNA in a stable state by enhancing m^6^A modifications to maintain the high expression level of DROSHA in BCSCs, which is primarily based on the ability of AURKU to inhibit the ubiquitination and degradation of METTL14 to stabilize DROSHA mRNA. AURKU also stabilizes m^6^A-mediated DROSHA mRNA by enhancing IGF2BP2 (146). The above studies suggest that the m^6^A modification is a potential target for BCSCs.

#### 4.1.3 m^6^A in GSCs

GBM is the most common and highly destructive primary malignant brain tumor. Recurrence is difficult to avoid, even with surgical resection and intensive radiation therapy. The median survival of patients with GBM is only one year (147). GBMs have significant heterogeneity both inside and outside of tumors. Usually, the cells at the top of the cell hierarchy have stem-like properties. GSCs are self-renewing, resistant to conventional therapy, and maintain long-term tumor growth, which is an important cause of tumor recurrence (148). Hence, studying the mechanisms underlying GSC self-renewal and proliferation could provide a better understanding of GBM tumorigenesis and help identify better therapeutic measures. The m^6^A modification is essential for GSC self-renewal and tumorigenesis. m^6^A demethylase ALKBH5 is highly expressed in GSCs, and the proliferation of patient-derived GSCs can be inhibited by silencing the expression of ALKBH5. Integrative transcriptome and m^6^A-seq analyses showed that ALKBH5 can demethylate the mRNA of the transcription factor FOXM1, thus leading to enhanced expression levels of FOXM1 and the recovery of tumor growth (149, 150).

Recent research progress has also been made on radiation resistance in GBM. ALKBH5, a recognized oncogenic factor, is abnormally elevated in expression in GBM. Previous studies have found that DNA damage repair is upregulated in GSCs (151), which may be one of the reasons for the development of resistance to radiotherapy, and showed that the sensitivity of GSCs to radiotherapy increased when *ALKBH5* was downregulated (152). ALKBH5 expression is blocked by ALKBH5 siRNA in GSCs, thus revealing that the expression of genes involved in homologous recombination repair was significantly decreased (152). The results validated by further experiments showed that blocking ALKBH5 could delay DNA damage repair (152) and suggested that inhibitors of ALKBH5 may be a potential therapeutic modality for overcoming resistance and improving sensitivity to radiotherapy.

Reports have also shown that the growth, self-renewal, and tumorigenesis of human GSCs are significantly promoted by knocking down key components of the RNA methyltransferase complex METTL3 or METTL14. In contrast, overexpression of METTL3 or inhibition of FTO activity inhibits the growth and self-renewal of GSCs. Interestingly, inhibition of FTO significantly delays tumor progression and extends the life span of GSC-transplanted mice. An m^6^A-RIP sequence analysis revealed that the RNA methyltransferases METTL3 and METTL14 significantly promoted the growth and self-renewal of GSCs by regulating the mRNA expression of the GBM-associated gene *ADAM19* (153).

Additional studies have confirmed that METTL3 can confer radioresistance to GSCs by enhancing SOX2-dependent DNA repair (154). Enhanced sensitivity of GBM to γ-irradiation is observed when *METTL3* is silenced, thus providing evidence for *METTL3* as a potential molecular target for GBM treatment (154). Moreover, METTL3 is also required to promote tumorigenesis by maintaining the expression of GSC-specific active transcriptional genes (155).

YTHDF1, a recognition protein of m^6^A, is also involved in GBM development. Aliaksandr et al. (156) found that tumorsphere formation, stemness marker expression, and migration capacity were decreased after knocking down *YTHDF1*. They concluded that *YTHDF1* is required to maintain CSCs in GBM cell lines. This study found that Musashi-1 (MSI1), an RNA-binding protein, is a marker of poor prognosis that can positively regulate the expression of YTHDF1. Thus, YTHDF1 and MSI1 are potential prognostic markers and therapeutic targets.

#### 4.1.4 m^6^A in CRSCs

The global cancer statistics report shows that CC is a common cancer that presented high incidence, death, morbidity, and mortality rates in 2021 (157). The mortality and morbidity of CC are rising in China, with 590,000 new cases and 300,000 deaths observed in 2021 (158). Despite improvements in medical care, the 5-year survival rate for patients with CC is only 64.9% (159). In recent years, numerous studies have shown that CSCs play an important role in CC (160–162). Therefore, investigating the molecular regulatory mechanisms of CRSCs in CC renewal and metastasis may be a potential approach to treating CC.

CBX8, also known as human polycomb 3, is significantly expressed in chemotherapy-resistant patients with CC. It is closely associated with stemness markers, such as LGR5, CD133, and CD44, in CC tissues. The RNA Modification Database (RMBase) showed that CBX8 mRNA is distributed with many high-confidence m^6^A sites. Therefore, researchers have investigated whether there is an association between m^6^A and CBX8 and queried The Cancer Genome Atlas database, and they showed that METTL3 is significantly upregulated in CC and METTL3-mediated m^6^A modification induces CBX8 overexpression and maintains the stemness of CC cells (163). This study provides a useful target for reversing the stemness of CC cells and improving the sensitivity of these cells to chemotherapy.

The expression of YTHDF1 is higher in CC than in normal tissues, whereas the expression of other YTH domain family members, YTHDF2, YTHDF3, YTHDC1, and YTHDC2, shows limited differences between colorectal tumors and normal tissues (164). Later studies showed that knocking down YTHDF1 inhibits the activity of the Wnt/β-catenin pathway in CC cells and significantly downregulates the expression of CRSC markers, thereby reducing the tumorigenicity of CRSCs *in vitro* and the tumor growth of mouse xenografts *in vivo* (165). Another study investigated the role of YTHDF1 in the Wnt/β-catenin protein signaling pathway in CC (166) and identified the checkpoint TCF7L2, which is activated by β-catenin. YTHDF1 promotes the translation of Wnt signaling effectors, including TCF7L2/TCF4, and activates Wnt signaling while enhancing β-linked protein activity. This process is required to maintain the stemness of intestinal stem cells. The above two studies suggest that the regulation of YTHDF1 plays a key role in the tumorigenicity and maintenance of CRSCs and thus may have revealed a potential therapeutic target for CC.

Zhao et al. (167) found that berberine has similar effects as FTO inhibitors. Reducing β-catenin can increase FTO in CRSCs and then reduce m^6^A, resulting in decreased stemness and increased sensitivity to chemotherapy. The study also suggested new strategies for developing new antitumor drugs that target Wnt/beta-catenin.

#### 4.1.5 m^6^A in HCSCs

Hepatocellular carcinoma (HCC) is a common pathological type of liver cancer and an important cause of death due to cancer. Advanced HCC is poorly treated and generally has a poor prognosis. For advanced HCC and recurrent HCC, effective treatment modalities are lacking. Therefore, understanding the molecular mechanisms of HCSCs may be an avenue to discover effective treatment modalities. YTHDF2 is required for the mRNA of OCT4 (pluripotency factor) to maintain m^6^A methylation at the 5′-UTR, which in turn promotes the expression of OCT4 protein and the formation of the HCSC phenotype (168). S-adenosylmethionine decarboxylase proenzyme has been identified as an oncoprotein. Recently, researchers have explored the role of S-adenosylmethionine decarboxylase proenzyme in HCC. They announced that increased levels of S-adenosylmethionine decarboxylase proenzyme could promote the expression of stemness genes such as *SOX2*, *KLF4*, and *NANOG* in HCSCs through FTO-mediated m^6^A demethylation in mRNA (169). All of the above studies provide ideas for targeting HCSCs.

#### 4.1.6 m^6^A and other solid CSCs

Recently, the role of m^6^A has also been studied in stem cells of other solid tumors, including lung cancer, pancreatic cancer, bladder cancer, osteosarcoma, ovarian cancer, oral squamous cell carcinoma, and cutaneous squamous cell carcinoma. For example, Liu et al. (170) found that ALKBH5 was highly expressed in CSCs derived from non-small cell lung cancer. p53 could regulate m^6^A levels by regulating ALKBH5, which in turn affected stemness gene expression in lung CSCs. Hu et al. (171) proclaimed that IGF2BP2 acts as a reader of m^6^A modification on lncRNA DANCR in pancreatic adenocarcinoma. Its upregulation stabilizes lncRNA DANCR and promotes stemness-like properties of pancreatic adenocarcinoma cells. Gao et al. (172) revealed that METTL3 and m^6^A modifications were significantly higher in bladder cancer CSCs than in non-CSCs. AFF4 binds to the promoters of *SOX2* (stemness gene) and *MYC* (oncogene) to activate their transcription. METTL3 promotes the self-renewal ability of BCSCs by regulating m^6^A levels in mRNA and the expression of AFE4. Wang et al. (173) obtained osteosarcoma stem cells with stemness characteristics from osteosarcoma cells treated with progressively increasing concentrations of chemotherapeutic agents. RNA-seq results of these cells showed that the mRNA methylation levels of genes that maintain stemness were significantly increased. They hypothesized that the occurrence of osteosarcoma stem cells is closely related to the maintenance of m^6^A modification. Huang et al. (174) revealed that FTO was underexpressed in ovarian cancer cells and ovarian CSCs. The self-renewal and tumor formation ability of ovarian CSCs was decreased after overexpression of FTO. Two phosphodiesterase genes (*PDE4B* and *PDE1C*) were identified as FTO targets. They play a key role in maintaining ovarian CSC stemness. Omprakash et al. (175) found that DDX3 (a human DEAD-box RNA helicase) reduced the m^6^A modification of mRNAs of two stemness genes (*FOXM1* and *NANOG*) through ALKBH5 in oral squamous carcinoma, resulting in reduced CSCs. Zhou et al. (176) discovered that the knockdown of METTL3 decreased the self-renewal ability of stem-like cells in cutaneous squamous cell carcinoma. These findings lead us to think that m^6^A might be related to stem cells in many other tumors. It also further affirms the relationship between m^6^A and CSCs and provides a direction for finding new therapeutic modalities.

### 4.2 Other RNA modifications in CSCs

**Figure 3:**
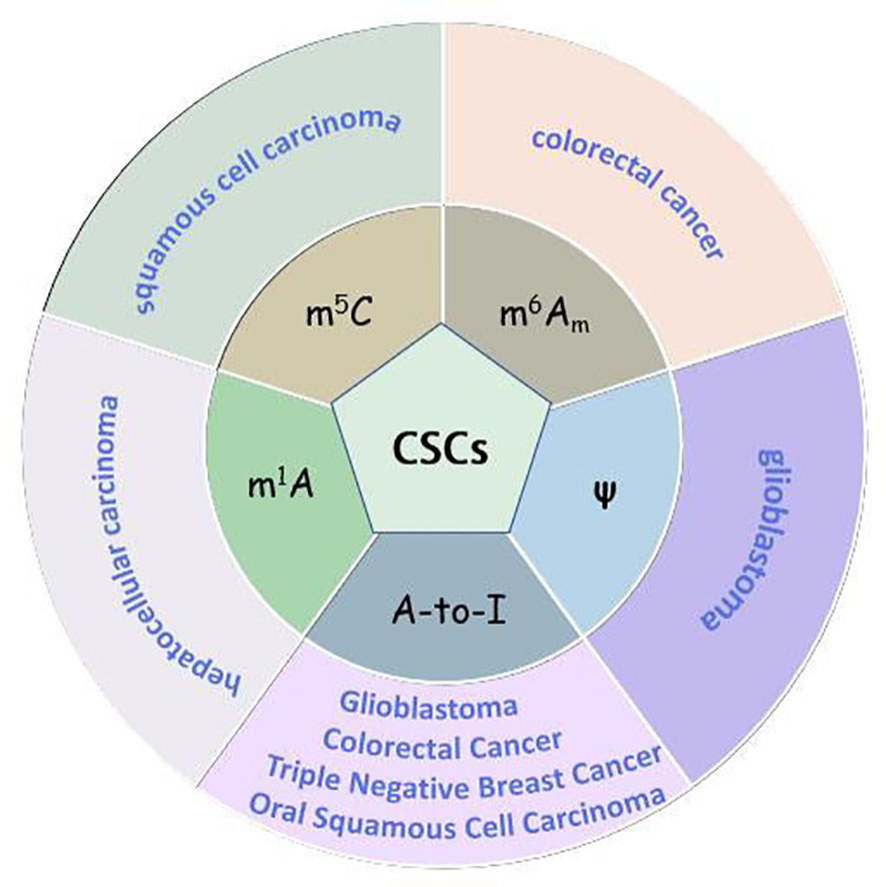
The role of m^5^C, m^1^A, m^6^A_m_, ψ, A-to-I in human CSCs.

More and more studies have found the relationship between CSCs and other RNA modifications. These findings are exhibited as follows (**Figure 3**).

#### 4.2.1 m^6^A_m_ in CSCs

Sun et al. [172] developed an m^6^A_m_-seq assay based on demethylation and RNA immunoprecipitation *in vitro*. This technique can directly differentiate m^6^A_m_ and 5′-UTR N6-m^6^A and thus aid in the in-depth study of the functions of both modifications (177). Unlike m^6^A, the function of m^6^A_m_ modifications is not well understood in mRNA. Recently, a study found that m^6^A_m_ levels were elevated in cell lines derived from patients with CC when FTO was expressed at low levels. Moreover, the tumorigenicity and chemical resistance of the cells are enhanced *in vivo* [173]. After a series of experimental validations, m^6^A_m_ was found to be a key transcriptomic marker controlling the stem-like cell phenotype in human CC cells. This deepened our understanding of the CSC phenotype and provided insights into developing a drug or therapy that reduces the ability of CSCs to metastasize and resist drugs (178).

#### 4.2.2 m^5^C in CSCs

m^5^C modifications play a variety of roles in various life processes, including self-renewal and differentiation of embryonic stem cells (179). Some studies have shown that m^5^C is closely associated with cancer pathogenesis, including initiation, metastasis, progression, recurrence, and drug resistance (180). However, there are very few studies on the relationship between m^5^C and CSCs. Blanco et al. (181) investigated the relationship between protein synthesis and cellular stress response and stem cell function and found that the writer of m^5^C, NSUN2, promotes protein synthesis by protecting tRNA from cleavage. When NSUN2 was inhibited, protein synthesis was decreased. Simultaneously, inhibition of NSUN2 function promoted stem cell function and tumorigenesis when posttranscriptional methylation was decreased in squamous cell carcinoma (181).

#### 4.2.3 ψ in CSCs

Ψ is the most abundant nucleotide modification in rRNA and tRNA and is also present in mammalian and yeast mRNA. It plays a role in RNA folding and secondary structure, stability, and translation. Ψ was the first identified RNA modification associated with cancer (35). PUS7 is a Ψ-depositing enzyme that promotes tumor growth. PUS7 is implicated in the formation and growth of CSCs. Ψ is one of the most abundant RNA modifications in GBM. Cui et al. (182) found that the growth of GSCs was inhibited when PUS7 expression was decreased in hormonal tumors. A specific PUS7-dependent Ψ modification was found in GSCs. In addition, they detected chemical inhibitors of PUS7 that inhibited PUS7-mediated Ψ modifications, suppressed tumorigenesis, and prolonged the lifespan of tumor-bearing mice (182). PUS7-mediated Ψ activates mTOG (mini-5´-terminal oligoguanine), which affects protein synthesis in HSCs. This driver mechanism can damage HSCs and may lead to marrow malignancies in humans (183).

#### 4.2.4 m^1^A in CSCs

Currently, m^1^A has been less studied in malignant tumors. Thus, the role of m^1^A methylation remains largely unknown in tumors. Studies on CSCs and m^1^A are even rarer. Intriguingly, Wang et al. (184) found that m^1^A modification of tRNA was significantly elevated in HCCs. The m^1^A methylation signal also became abnormally upregulated in HCSCs. Therefore, they explored the mechanism and showed that TRMT6/TRMT61A-mediated m^1^A enhanced PPARδ (peroxisome proliferator-activated receptor δ) protein translation. PPARδ could promote self-renewal and tumor growth in HCSCs.

#### 4.2.5 A-to-I in CSCs

Because A-to-I is abundant in the human brain, it has been more extensively studied in the nervous system. Decreased ADAR2 is closely associated with the recurrence and aggressiveness of glioma (98, 99). In addition, ADARs play oncogenic roles in malignant tumors, such as liver, bladder, and lung cancers, through different mechanisms (100–102). However, a small number of studies has shown that ADAR1 plays a role in tumor suppressor genes (103). In conclusion, studies on A-to-I in cancers are gradually increasing, and they revealed that A-to-I is an important mechanism for cancer development and an important target for cancer therapy.

The relationship between A-to-I and cancer stemness has also been reported. For example, Liu et al. (185) demonstrated that ADAR1 oral squamous cell carcinoma is closely associated with stemness. When *ADAR1* is knocked down, the expression of SOX2 and POUF51 is reduced, as shown by a protein blotting assay, and the stemness of oral squamous cell carcinoma lines was also found to be attenuated. These studies suggest that ADAR1 can maintain the stemness phenotype of oral squamous cell carcinoma. GSCs are an important factor that contributes to recurrence and drug resistance in GBM. Li et al. (186) found that ADAR1 expression was increased in GSCs. The TYK2/ADAR1/GM2A axis was found to be critical in maintaining the stemness of GSCs; thus, it is likely to be a new clinical strategy for GBM treatment. Lee et al. (187) explored the transcriptome and epitranscriptome landscape of triple-negative breast cancer (TNBC) and identified CSC-like microecological sites in TNBC containing characteristic A-to-I editing. In addition, *ADAR1*-mediated A-to-I editing leads to an increase in AZIN1 expression, thereby enhancing the stemness of colorectal adenocarcinoma cells (188).

## 5 Conclusion and future scope

In recent years, technological breakthroughs have rapidly increased our understanding of the mechanisms of RNA modifications. The possible role of posttranscriptional modifications in human diseases has been revealed and recognized. Nevertheless, the epitranscriptome is complex and diverse, and we have probably touched only the tip of the iceberg in terms of its study. Although many RNA modifications contributions to carcinogenesis have been identified, their molecular targets in cancer pathogenesis have not been revealed. Moreover, abnormal expression of RNA modifying enzymes has been found in many cancer cells, but how specific RNA modifications affect different cancer cell subpopulations remains largely unknown. Thus, the field of epitranscriptomics requires comprehensive development, including the creation of new assays, the improvement of assay sensitivity, and the development of detailed research strategies. The detection of RNA modifications by next generation sequencing (NGS) requires selective chemical treatment or antibody immunoprecipitation for specific modification types. At the same time, these methods also face the problems of short reading length, isomer ambiguity, bias and artefacts. Direct RNA sequencing (DRS), commercialized by Oxford Nanopore technologies (ONT), is able to directly detect any given modifications in a single transcript, improving these limitations of NGS-based methods. For example, Zhang et al. (189) presented DirectRMDB, the first ONT-based quantitative mapping database of RNA modifications, which includes 16 types and a total of 904,712 modification sites in 25 species from 39 independent studies. This platform integrates three generation sequencing and eight RNA modification detection technologies, making it possible to easily query specific modifications on RNA isomers.

In addition, thanks to advances in high-throughput sequencing techniques for transcriptome analysis, the emergence of large databases of RNA modifications has become an exciting field that promotes further research into the mechanisms and functions of these RNA modifications. By using machine learning methods, the localization of RNA methylation sites in humans, mice and other species has been predicted more accurately. At the same time, new computational methods are being developed to understand the disease associations of individual RNA methylation sites by utilizing the large amount of apparent transcriptome data accumulated in existing studies (190). Such as DRUM, an online database that incorporates the associations among diseases, genes and m^6^A RNA methylation sites from gene expression, RNA methylation and disease similarities data with the Random Walk with Restart (RWR) algorithm, was used to query the top m^6^A sites related to 705 different diseases (191). As a comprehensive database, m6AVar enables systematic association analysis to identify associated variants that may affect RBP binding regions, miRNA targets, and splicing sites. m6AVar can also be used to identify pathogenic variant modifications that cause m^6^A dysregulation (192). To fully uncover the association between disease-related variants and their epigenetic transcriptome disturbances, Chen et al. established RMDisease, a database of genetic variants that correlate RNA modifications with underlying diseases (193). Recently, the authors have updated RMDisease to RMDiseasev2.0 by collecting all available RM-related variants and annotating their potential participation in diseases and traits (194). Song et al. proposed a comprehensive online platform, m6A-TSHub, to reveal m^6^A methylation and m^6^A-relateded mutations in 23 human tissues. This is particularly important for studying the epigenetic transcriptome of diseases associated with specific tissues, especially cancer-related diseases (195). As high-throughput apparent transcriptome data becomes more available, advanced techniques for RM analysis are being developed, and databases linking RNA modification to disease, particularly tumor disease, are being developed to better understand and explore the potential association between modification and tumor disease.

Given that RNA modifications play an important role in a variety of cancers, the use of RNA modifications as diagnostic and prognostic indicators or effective therapeutic strategies has great potential for clinical application. Several current studies related to the link between RNA-modifying enzymes and cancer have also provided a basis for the rational clinical design of efficient and specific RNA inhibitors. For example, through structural and biochemical analysis, the natural product rhein was found to competitively bind to the active site of FTO, thereby preventing FTO from binding to the m6A substrate (196). Therefore, it has an inhibitory effect on m6A demethylation activity (196). Another FTO inhibitor, meclofenamic acid, is an FDA-approved NSAID that also competes with FTO for binding and thus increases m6A levels in mRNAs in cells (197). The successful inhibition of the *in vitro* and *in vivo* growth of GSCs by meclofenamic acid 2, an ethylester derivative of meclofenamic acid, was recently reported (146). However, according to current findings, RNA modifications sometimes act on proto-oncogenes or oncogenes. Because of the contradictory role of epitranscriptomics, drug studies should be conducted with great caution to ensure safety. Since RNA-modifying enzymes are involved in a large number of life processes, the effect of small molecule agents on other normal life processes should be taken very seriously when treating tumors. For example, in the absence of RNA methylesterase, the affected organs are usually the brain and testis (198, 199).

With advances in sequencing technology and deciphering of the human genome, our understanding of cancer has changed substantially in the past decade. RNA modifications have been found to play an important role in carcinogenesis and imparts stemness to cancer cell subpopulations. On the one hand, RNA modifications can promote cancer progression by reducing the stability of cancer suppressor genes to eliminate their inhibitory effect. On the other hand, they can enhance the stability and expression of the proto-oncogene transcripts. Cancer genomes have been revealed as complex and heterogeneous, and carcinomas do not arise from a single clone with a single tumor genome. CSCs are a group of cells with self-renewal capabilities that drive tumor growth and reconstitute tumor heterogeneity. CSCs are in a static state during treatment and have strong resistance to treatment; thus, they represent an important cause of tumor recurrence, metastasis, and drug resistance. RNA modifications are closely related to CSC formation and stemness maintenance. In this paper, we discuss recent advances in the relationship between RNA modifications and cancer stemness and the impact of these modifications on cancer formation. The development of new targeted drugs based on the effects of RNA modifications on CSCs represents a very promising treatment direction. Moreover, because of the different abnormal signaling pathways within CSCs, combining drugs from other targets to eradicate CSCs is a promising therapeutic modality. Traditional treatment modalities for cancers mainly target non-CSCs in tumor tissues. Traditional tumor therapy combined with CSC-targeted therapy can kill a large number of tumor cells while eliminating CSCs, thus causing metastasis and recurrence, and it also represents a therapeutic direction that should be further explored.

## Author contributions

All authors have contributed to this review. JG has provided the conception. LH has written the first and revised draft. JZ, ZL,XL have revised the draft. All authors contributed to the article and approved the submitted version.
